# Roles of RIPK3 in necroptosis, cell signaling, and disease

**DOI:** 10.1038/s12276-022-00868-z

**Published:** 2022-10-12

**Authors:** Michael J. Morgan, You-Sun Kim

**Affiliations:** 1 Department of Natural Sciences, Northeastern State University, Tahlequah, OK 74464 USA; 2 Department of Biochemistry, Ajou University, School of Medicine, Ajou University, Suwon, 16499 Korea; 3 Department of Biomedical Sciences, Graduate School, Ajou University, Suwon, 16499 Korea

**Keywords:** Inflammatory diseases, Necroptosis

## Abstract

Receptor-interacting protein kinase-3 (RIPK3, or RIP3) is an essential protein in the “programmed” and “regulated” cell death pathway called necroptosis. Necroptosis is activated by the death receptor ligands and pattern recognition receptors of the innate immune system, and the findings of many reports have suggested that necroptosis is highly significant in health and human disease. This significance is largely because necroptosis is distinguished from other modes of cell death, especially apoptosis, in that it is highly proinflammatory given that cell membrane integrity is lost, triggering the activation of the immune system and inflammation. Here, we discuss the roles of RIPK3 in cell signaling, along with its role in necroptosis and various pathways that trigger RIPK3 activation and cell death. Lastly, we consider pathological situations in which RIPK3/necroptosis may play a role.

## Cell death: Multiple roles for enzyme

Further research into the various roles of an enzyme involved in necroptosis, a form of programmed cell death, could present novel therapeutics for multiple diseases. Necroptosis involves the rupture of cellular membranes in response to stress, for example when the body is under attack from pathogens. The contents of the cell spill out, triggering a proinflammatory immune response. You-Sun Kim at Ajou University in Suwon, South Korea, and Michael Morgan at Northeastern State University in Tahlequah, USA, reviewed the activity of receptor-interacting protein kinase 3 (RIPK3) in cell signaling and necroptosis. In initiating necroptosis, RIPK3 forms a complex with another enzyme, which then activates a protein involved in regulating membrane permeability. Disruption of necroptosis is implicated in inflammatory and neurodegenerative diseases, cancers, and conditions involving tissue damage such as heart disease.

## Introduction

The finding that cell death is a genetically encoded and evolutionarily conserved process in multicellular organisms was a highly significant discovery at the end of the last century; its discoverers were awarded the 2002 Nobel Prize for Physiology and Medicine^1^. Typically referred to as “programmed cell death” (PCD) in cases where it is initiated in the case of a physiological setting (e.g., development) or “regulated cell death” (RCD) in cases where the program is initiated by an external stimulus (e.g., chemotherapy)^2^, the processes by which cells commit “cell suicide” are of vast and profound importance to the normal physiology of living things and to our ability to intervene in pathological situations. PCD allows tissues and organs to be shaped and organized during development and continues to play roles in adults, where it functions in tissue remodeling, organ and tissue homeostasis, immunity, and tumor suppression, among many other processes. Whereas coordinated PCD contributes to health homeostasis, inadequate PCD or overactivation of PCD frequently leads to pathological conditions and disease. While cell death happens through a number of mechanisms or modes, “apoptosis” is considered to be the most physiological form, and is primarily executed through the activation of cysteine proteases of the caspase family. These proteases target proteins involved in the cell cycle and DNA repair, structural proteins, transcription and survival factors, and other regulatory proteins, thus leading to the organized destruction of the cell.

In the initial studies highlighting its discovery, receptor-interacting protein kinase-3 (RIPK3, also referred to as RIP3) was proposed to be a regulator of apoptosis downstream of the tumor necrosis factor (TNF) receptor 1 signaling complex since its overexpression led to caspase activation and cell death^3–5^. This proposal was early in the cell death field, and a few years after the discovery that ligands of the TNF superfamily were capable of inducing the caspase-dependent apoptotic pathway, it became clear that alternative cell death pathways were also initiated in the absence of caspase activation that led to a form of death with a “necrotic-like” morphology^6–10^ that was later referred to as “necroptosis”^11^. For a number of years, receptor-interacting protein kinase-1 (RIPK1, also referred to as RIP1) was the only downstream factor known to be involved in necroptosis^8,12^. Almost a decade after the discovery of RIPK1, RIPK3 came to the forefront of necroptosis studies when it was determined that it interacts with RIPK1 during necroptosis and is an essential downstream partner for RIPK1 for this form of death^13–15^. The kinase-dependent role of RIPK3 in necroptosis is now considered its prototypical role in cellular function; however, it is now clear that RIPK3 plays several different roles in cells and perhaps has more than one function within necroptosis itself.

## RIPK3 in necroptosis

To clarify the role of RIPK3 in necroptosis, let us first clarify the important differences between apoptosis and necroptosis; furthermore, since some facets of necroptosis resemble those of classic necrosis, let us also clarify how necroptosis differs from necrosis. As mentioned, the downstream consequence of apoptosis is the activation of the caspase proteases that cleave their different substrates to trigger cell death. The nature of these cleavage events results in a very organized process of cell death. Among the characteristics of this cell death are cellular shrinkage, chromatin condensation and fragmentation, nuclear condensation, and fragmentation, concluding in the formation of membrane blebs that break off and become membrane-bounded bodies that are rapidly phagocytosed by surrounding cells and professional phagocytes of the immune system. Because of this mechanism of action, apoptosis limits cell debris and content leakage that would trigger inflammation.

In contrast, classic necrosis is a passive, nonprogrammed form of cell death that is not genetically encoded but is the result of direct cellular injury or other pathological trauma. As a passive form of cell death, it requires no energy, and it is characterized by the swelling of cells and/or organelles rather than shrinkage. Necrosis, therefore, results in (or is directly caused by) plasma membrane rupture, thus cellular contents are leaked, and inflammation is triggered^16,17^.

Necroptosis, on the other hand, is a mixture of apoptosis and necrosis. Like apoptosis, specific gene products are required for necroptosis, and the cell itself has a central role in initiating its own demise through a cellular “program”. Some energy is required for necroptosis, as kinase activity is essential. Like necrosis, necroptotic cell death results in plasma membrane permeability, cell leakage, and immune system activation. Thus, apoptosis, which has been thought to occur primarily without triggering inflammation (this is, in fact, an oversimplification, as in some contexts, apoptosis also activates the immune system), is largely perceived as having different physiological outcomes than necroptosis, which is highly proinflammatory^18,19^. In addition, necroptosis processes have more recently been shown to activate the immune system somewhat differently than classic necrosis. Necroptotic cells may play multiple roles in innate immunity and shape subsequent adaptive immunity through the release of endogenous danger signals known as damage-associated molecular patterns (DAMPs)^20,21^, which interact with pattern recognition receptors (PRRs) of innate immune cells to prime immune cells to respond to pathogens and potentially harmful cells, such as those that are infected or tumorigenic. The de novo synthesis of cytokines and chemokines occurs especially in dying necroptotic cells^22–24^, likely due in part to the types of signals that trigger necroptosis. Indeed, the activation of RIPK1/RIPK3 leads to the upregulation of inflammatory chemokines to promote the cross-priming of CD8^+^ T cells^25–27^ and/or promote the release of DAMPs^28^; thus, necroptosis is believed to significantly contribute to antitumor immunity.

As mentioned, necroptosis is highly dependent on RIPK3 as an essential part of the necroptotic machinery^13–15^. The RIPK3 protein is characterized by an N-terminal kinase domain, with which it phosphorylates itself and other substrates, and a C-terminal domain that contains a receptor-interacting protein homotypic interaction motif (RHIM) through which it associates with other proteins to oligomerize. Once activated (as described below), RIPK3 autophosphorylates and then phosphorylates and activates a pseudokinase called MLKL^29,30^, which is essential for membrane permeabilization during necroptosis^31,32^. The phosphorylation of MLKL by RIPK3 causes a conformational change in the MLKL protein that exposes its N-terminal four- or five-helical bundle domain that is usually tightly bound to the pseudokinase domain^32,33^, but that once released from its interaction with the pseudokinase domain mediates MLKL oligomerization. Reports have significantly varied as to how many MLKL subunits (3, 4, 6, 8, or more) are involved in the oligomers^34^, perhaps because of differences between the mouse and human systems^35,36^, or perhaps because the number of subunits may vary between where it is activated and where it is inserted^37^. The oligomerization of MLKL promotes its membrane translocation, which is followed by membrane permeabilization. The exact mechanism for this permeabilization has been debated, but MLKL has been reported to bind to phosphoinositides^38–41^ and cause membrane leakage, perhaps through the formation of channels or pores^37,42,43^ or indirectly through interaction with ion channels that let various cations through^44,45^. Regardless of whether this involves further osmotic pressure, the membrane is sufficiently permeabilized to let cellular contents out and kill the cell.

## Activation of RIPK3 by multiple stimuli

**Fig. 1:**
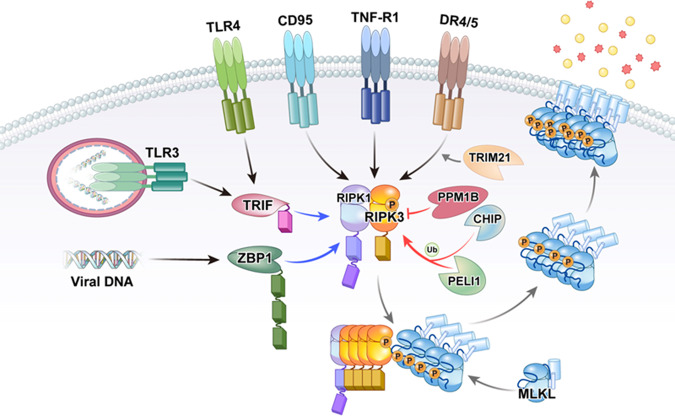
Activation of RIPK3 by multiple stimuli. RIPK3 can be activated via various receptors when bound by their respective ligands. These are TNF receptor 1 (TNF-R1), CD95, death receptors (DR4/5), Toll-like receptors (TLR3/4), and Z-DNA-binding protein-1 (ZBP1)/DAI. In the first three of these pathways (but not TLR3/4 or ZBP1), RIPK1 is required and binds to RIPK3 through its receptor-interacting protein homotypic interaction motif (RHIM). In the case of ZBP1, RIPK3 is recruited directly via the ZBP1 RHIM domain, while in the case of TLR3/4, RIPK3 is recruited indirectly via the RHIM domain of TRIF. Once activated, RIPK3 autophosphorylates and then phosphorylates and activates MLKL to induce a conformational change and translocation to the membrane, where membrane permeabilization follows. During this process, post-translational modifications positively and negatively regulate the necroptosis pathway. Two E3 ligases, Pellino-1 (PELI1) and carboxy terminus of HSC70-interacting protein (CHIP), may control the basal threshold of necroptosis. Another E3 ubiquitin ligase, TRIM21, is proposed to be a regulator of necroptotic cell death in response to TRAIL. PPM1B suppresses necroptosis by dephosphorylating RIPK3.

Necroptosis is initiated downstream of many cellular stressors, including the signaling events activated by death receptor ligands, such as TNF-α, FasL, or TRAIL, that act through their various death receptors^17,46^. This is where necroptosis was discovered and where most research has been conducted. In actuality, the term “necroptosis” was initially applied specifically only to nonapoptotic death receptor-initiated cell death^11^ but was then redefined to include any cell death process “that critically depends on MLKL and on the kinase activity of RIPK1 (in some settings) and RIPK3”^47^. Since the discovery of MLKL, many cell death stimuli have been added as initiators of necroptosis. Almost all of these can be classified as pattern recognition receptors (PRRs) of the innate immune system (see Fig. 1). These include Toll-like receptors 3 and 4 (TLR3 and TLR4, respectively) and ZBP1 (or DAI). Other pattern recognition receptors, such as retinoic acid-inducible gene I (RIG-I) (also referred to as DExD/H-box helicase 58, or DDX58), interferon-α and interferon-β receptor (INFAR1), and STING1, may induce necroptosis as well but are thought to initiate necroptosis indirectly through gene induction of one of the previously mentioned receptors^48–50^.

Necroptosis typically, but does not always, involves the sister kinase of RIPK3, RIPK1, which is required for many necroptotic signals, such as the prototypical necroptotic signaling pathway downstream of death receptors that are initiated by TNF-α through TNFRSF1B (TNF receptor 1)^8,12^. RIPK3 interacts with RIPK1 through its receptor-interacting protein homotypic interaction motif (RHIM)^51^. Although RIPK1 is a kinase, most of its signaling pathways do not actually require its kinase activity^52^ but rather its function as a scaffolding protein. An exception to this is its signaling role in necroptosis. The assembly and activation of the RIPK1-RIPK3 complex downstream of TNF-α are dependent on the activities of both of these kinases^13,53^ to autophosphorylate themselves but not apparently to phosphorylate each other^54^. This complex, with its associated proteins, including FADD and caspase-8, is often referred to as the necrosome. The downstream RIPK1-RIPK3 complex is believed to form a large amyloid-type aggregate through the interaction of the two proteins^55,56^, although it is probably the oligomerization of the proteins and not the amyloid nature of the complex that leads to necroptosis^57^. When caspase-8 activity is high, apoptosis prevails by several mechanisms of action. Firstly, active caspase-8 activates downstream apoptosis factors, but secondly, caspase-8 and other caspases cleave proteins that are essential for necroptosis, including RIPK1^58^, RIPK3^59^, and the CYLD deubiquitinase^60^ (the last of which potentiates necroptosis by removing ubiquitin from RIPK1, allowing it to interact with RIPK3), thus stabilizing RIPK1/RIPK3 oligomerization and downstream activation of MLKL. As RIPK1 plays many important nonnecrotic proinflammatory and survival roles in death receptor signaling, the prosurvival roles of RIPK1, as well as its apoptotic roles, often must be prevented for necroptosis to proceed. For instance, in the TNF-α pathway, RIPK1 is essential for the efficient activation of the prosurvival transcription factor NF-κB, as well as the MAP kinases ERK, JNK, and p38^61^, which sometimes may also result in prosurvival signals. RIPK1 not only positively regulates the activity of the necrosome complex after necrotic stimuli but also negatively regulates promiscuous basal RIPK3 induction of necrotic cell death^57,62–64^.

Thus, there are generally two main conditions that are important for necroptosis to prevail downstream of death receptors: a significant reduction in caspase activity and the inhibition of the various E3 ubiquitin ligases and other proteins that ubiquitinate or otherwise modify RIPK1 and drive it toward the induction of other pathways to prevent it from associating with RIPK3.

The importance of apoptotic regulation of necroptosis is highlighted by the knowledge that the developmental defects/lethality of apoptotic gene deletions, including FADD knockout, caspase-8 knockout, cFLIP-FADD double knockout (but not cFLIP knockout alone), XIAP-cIAP1 double knockout and cIAP1-cIAP2 double knockout, are rescued completely or to some degree by RIPK1/RIPK3 deficiency^65–72^.

### Activation of necroptosis by TNF-α through TNFRSF1B (TNF receptor 1, or TNF-R1)

The activation of necroptosis, as mentioned previously, involves RIPK1. Signaling within the TNF-R1 complex is mediated in large part by the recruitment of the death domain-containing proteins TRADD and RIPK1, which bind (via their death domains) to each other and to the internal death domain of the TNF-R1 receptor upon activation by TNF^46,61^. RIPK1 can be recruited to TNF-R1 in the absence of TRADD, especially when cells express high levels of RIPK1^73–76^; however, most studies show that RIPK1 ubiquitination is lost in the absence of TRADD. Some studies have found a significant reduction in RIPK1 recruitment to the activated TNF receptor in TRADD knockout cells^73,74^, while other studies found no difference in the recruitment of unmodified RIPK1 in its absence^75–77^. Once recruited to the complex, RIPK1 is modified by several post-translational modifications, including phosphorylation and polyubiquitination, through several mechanisms. K63 ubiquitination of TRADD recruits TRAF2, which then recruits RING finger E3 ligases cellular inhibitor of apoptosis protein-1 (cIAP1) and cIAP2, which promote K63 ubiquitination of RIPK1 on its internal domain. Further recruitment of the linear ubiquitin chain assembly complex (LUBAC) promotes linear M1 polyubiquitination.

The modification of RIPK1 via K63 allows the recruitment of the TGFβ activated kinase-1 (TAK1) and IκB kinase (IKK) complexes, which results in the activation of ERK, JNK, p38 and NF-κB^78^. A20^79^, CYLD^80^, and OTULIN^81^ ubiquitin hydrolases remove the K63-linked and linear ubiquitination of RIPK1. Other polyubiquitination events modify RIPK1 with K48-linked ubiquitin chains that promote the proteasomal degradation of RIPK1^82,83^. While RIPK1 degradation would in itself downregulate cell death, K48-modified RIPK1 is also unable to trigger necroptosis. Correspondingly, some phosphorylation events promote the cell death activity of RIPK1, while other phosphorylation events (for instance, Ser25 phosphorylation by IKKs^84^ or Thr189 phosphorylation by TBK1^85^) inhibit cell death and necroptosis.

Whilst RIPK1 is associated with the TNF receptor I complex, it functions in cell signaling events, but does not appear to be involved in cell death. However, should RIPK1 lose its protective phosphorylation and polyubiquitination, it dissociates from the main complex and forms secondary complexes, with or without TRADD. It is believed that secondary TRADD-dependent complexes induce apoptosis independent of RIPK1 or its kinase activity, while complexes with RIPK1, FADD, and caspase-8 initiate apoptosis that is dependent on the kinase activity of RIPK1^86,87^. Although FADD and caspase-8 are in the secondary complexes, these are not required for TNF-dependent necroptosis but are both inhibitory of the necroptotic process^77^, in part through caspase-dependent cleavage of RIPK1 and RIPK3, as has been mentioned^58,59^. Assuming that RIPK1 is not inactivated by caspase-8^58^, the autophosphorylation of RIPK1 leads to the association of its RHIM domain with that of the RHIM domain of RIPK3^51,57^, and the oligomerization of these components leads to the active necrosome^13–15^, which resembles an amyloid fiber^55^, and mediates the phosphorylation of MLKL that is required for necroptotic cell death^29–32^. In contrast to when in the receptor complex, two different K63 ubiquitination events that occur to RIPK1 later actually promote necroptosis by promoting necrosome formation. The E3 ligase c-Cbl promotes K63-linked polyubiquitination of RIPK1 under conditions where TAK1 is inhibited, leading to a detergent-insoluble aggregation of RIPK1 and its binding partners and stimulating necroptosis^88^. Pellino-1 (PELI1) mediates K63-linked polyubiquitination to kinase-active RIPK1, causing it to more strongly bind and activate RIPK3^89^. Conversely, ubiquitination by the carboxy terminus of HSC70-interacting protein (CHIP) leads to lysosomal degradation of the cytosolic, nonactivated pool of RIPK1^90^.

Curiously, these last two E3 ligases also control the negative regulation of RIPK3 through ubiquitination. While CHIP appears to downregulate the basal levels of RIPK3 (also through lysosomal-dependent degradation)^90^, PELI1 downregulates kinase-active RIPK3 that has already been activated in the necrosome through K48 polyubiquitination and proteasomal degradation^91,92^. Thus, these two E3 ligases may control the basal threshold of necroptosis in the cell. Other proteins are known to negatively control the RIPK1-RIPK3 interaction by interacting with one of the RIPK proteins. Among these are c-Myc (MYC)^93^ and Aurora kinase A (AURKA) and its substrate glycogen synthase kinase-3 beta (GSK3β)^94^.

Last, there are some other mechanisms by which the RIPK1-RIPK3-MLKL complex is controlled. For instance, protein phosphatase 1B (PPM1B) suppresses necroptosis by dephosphorylating RIPK3, which then prevents MLKL from being recruited to the necrosome^95^. Casein kinase family members, on the other hand, are known to reinforce the phosphorylation of serine 227^96,97^, which is the same event that initially occurs via autophosphorylation, and therefore promotes RIPK1-RIPK3-MLKL complex activation. Finally, reactive oxygen species (ROS), perhaps including those directly induced in the TNF receptor complex via NADPH oxidases^61,98,99^ or downstream of RIPK3^15,29^ or from the mitochondria^12,100–102^, may affect the stability of RIPK1-RIPK3-MLKL, although ROS, especially mitochondrial ROS, may not be absolutely required for necroptosis to occur^14,103^. It is proposed, for instance, that RIPK1 autophosphorylation is upregulated by ROS^104^. Given that the thiol groups of cysteine residues within the active sites of enzymes are often reactive with ROS due to their low pKA^105^, which is well established for inactivating not only classic protein tyrosine phosphatases^106–108^ but also dual specificity phosphatases^109^, it is likely that ROS may amplify necrosome formation by inactivating phosphates that would remove the important activating phosphates on RIPK1, RIPK3, and MLKL. For instance, if PPM1B, which was mentioned above, was inactivated, RIPK3 would have a higher propensity to remain phosphorylated and to therefore activate MLKL.

### Activation of necroptosis by Fas ligand (FASLG) through CD95 (FAS)

FAS was the first receptor discovered to mediate RIPK1-dependent necroptosis^8^. Shortly after this, however, the Fas system was largely abandoned for studying necroptosis in favor of using the TNF system as a model for studying necroptosis. A few studies have employed FAS as a control or second model system for the verification of necroptotic requirements [see, for instance, refs. ^11,14^], but fewer actual mechanistic necroptosis studies have been performed using FAS than TNF-α. Therefore, many things about FAS-induced necroptosis have been largely inferred from the understanding of its well-known mechanisms for inducing apoptosis and comparing this with what is known about the TNF mechanism. The mechanism of FAS-induced necroptosis is believed to be somewhat similar to that induced through TNF-R1.

Although secondary complexes also occur in response to FASLG, it is not completely clear whether secondary complexes are essential for FASLG-induced necroptosis, since FADD and RIPK1 are recruited via their death domains directly to the cytoplasmic death domain of FAS, and caspase-8 is brought along directly into the receptor signaling complex^16^. Unlike the TNF pathway, FADD appears to be required for necroptosis in the FAS pathway, as FADD-deficient cells are completely resistant to both apoptotic and necroptotic cell death^8,110,111^. This might be because FADD supports RIPK1 recruitment to the complex. Similar to the TNF pathway, the inhibition of caspase-8 and cIAPs is usually required to direct the pathway away from apoptosis to necroptosis^8,68,71,112–114^. While little else is known about FAS-induced necroptosis (other than RIPK1 and RIPK3 are known to be essential for the process), it is assumed that necrosome function in FASLG-induced complexes functions downstream similarly to TNF-induced complexes, with RIPK1-RIPK3 oligomerization leading to MLKL phosphorylation.

### Activation of necroptosis by TRAIL (TNFSF10) through DR4 (TNFRSF10A) and DR5 (TNFRSF10B)

TRAIL-induced cell death is mediated by different receptors, but TRAIL also initiates necroptosis upon cIAP inhibition^14,115^ or TAK1 deficiency^115–117^ and/or when apoptosis is blocked. While two receptors, DR4 and DR5, mediate TRAIL signaling in the human system, only a single TRAIL receptor exists in mice, which appears to be more similar to DR5 than to DR4. TRAIL-initiated necroptosis is predicted to be very similar to the FASLG-induced necroptotic pathway, given that (as in FAS signaling) FADD is essential for necroptosis to proceed because it is largely through FADD-dependent mechanisms that complex components are recruited to the receptor^77,118–120^. RIPK1 is likewise essential for TRAIL-induced necroptosis^77,121,122^, but unlike the mechanisms involved in the FAS, TNF-R1, and DR5 receptors, RIPK1 does not directly interact with the receptor but is recruited through interactions with FADD-recruited caspase-8^123^, the FADD death domain itself, and possibly the FADD-recruited TRADD death domain^124^. Unlike in FAS signaling, TRADD is also recruited to the TRAIL receptor complex via FADD^124,125^, but in this case, it largely has a negative effect on cell death (at least upon apoptosis, necroptosis was not examined), possibly by reducing FADD recruitment to the receptor^124^. Alternatively, TRADD may promote survival signaling through additional recruitment of TRAF2. Curiously, although less FADD is recruited to the receptor, more RIPK1 is recruited to the receptor complex in the presence of TRADD than in its absence^124^. Similar to the TNF-R1 complex, ubiquitination negatively regulates necroptosis; for instance, linear ubiquitination of RIPK1 by receptor-recruited LUBAC blocks TRAIL-induced necroptosis^126^. Similar to c-Cbl in TNF-initiated necroptosis, the E3 ubiquitin ligase TRIM21 is an upregulator of necroptotic cell death in response to TRAIL^127^. As with FAS and TNF-R1 signaling, secondary complexes form downstream of the TRAIL receptor complex^128,129^; however, whether these complexes are required for necroptosis and whether the main receptor complex is capable of mediating necroptosis have not been studied.

### Activation of necroptosis by TLR4

The next best-studied pathway that induces necroptosis is probably that which is downstream of TLR4, which is activated in immune cells in response to bacterial lipopolysaccharide (LPS). RIPK3 is required for LPS-mediated necroptosis; however, although RIPK1 is recruited to some of the activated complexes, it does not appear to be required^130,131^ Rather, RIPK3 is recruited to Toll receptors through the cytosolic adaptor Toll/IL-1 receptor domain-containing adaptor protein inducing interferon-β (TICAM-1, also referred to as TRIF). This is one of two main adaptors that are recruited to TLR4, the other being myeloid differentiation primary response protein 88 (MYD88). TRIF contains a RIP homotypic interaction motif (RHIM) similar to RIPK1, by which it interacts with RIPK3. Thus, the TLR4 necrosome components downstream of TRIF are TRIF itself, RIPK3, and MLKL. Like RIPK1, TRIF is cleaved by active caspase-8^132^, allowing apoptosis to downregulate necroptosis in this context, and although TLR4 is not a potent mediator of caspase activation by itself, other pathways that are induced downstream, such as TNF, can activate caspase-8. For example, TLR3, which also utilizes TRIF, IS a strong inducer of caspase-8 activation. Likewise, cIAPs 1 and 2 limits the necroptosis induced by LPS, similarly to the TNF pathway^133^. In addition to the noncanonical activation of necroptosis that occurs downstream of TRIF, the MYD88 arm of the TLR4 pathway can also induce the canonical RIPK1/RIPK3/MLKL necrosome, although this may be dependent on the induction of TNF and the TNF-R1 pathway^134–136^.

### Activation of necroptosis by TLR3

TLR3 is a pattern recognition receptor that recognizes double-stranded (ds)RNA, such as poly(I:C), as well as UVB-damaged self-RNA^137^. TLR3 can induce apoptosis, which, like the other receptors mentioned, is negatively modulated by cIAPs^138^. Apoptosis requires RIPK1-mediated recruitment of FADD and caspase-8^139^. Necroptosis induced by this receptor is mediated by TRIF and requires RIPK3 and MLKL^130,136^. TLR3-dependent necroptosis does not require RIPK1 in most cells. However, there are clearly some cell-type differences in TLR3 signaling, as macrophages (but not fibroblasts or endothelial cells) require RIPK1 for TLR3-mediated necroptosis^136^.

### Activation of necroptosis by ZBP1 (DAI)

ZBP1/DAI is a nucleic acid pattern recognition receptor that binds and detects zDNA and zRNA from pathogens and induces necroptosis and apoptosis^140–143^. ZBP1 recruits RIPK3 via its RHIM domain^141^, which then recruits and activates MLKL to induce necroptosis^143^. RIPK1 is not essential for necroptosis induction through ZBP1 but actually inhibits both apoptosis and necroptosis induced by the receptor^144,145^.

## Nonnecroptosis roles of RIPK3

### Contributions to apoptosis-caspase-8

While RIPK3 is not considered an essential molecule for death receptor apoptosis, RIPK3 is required for the full initiation of caspase-8 activity when LPS-treated macrophages are treated with IAP inhibitors^146^. Moreover, while not absolutely essential, the presence of RIPK3 does contribute to TNF-induced apoptotic cell death under conditions of cIAP1/2 depletion or TAK1 inhibition^115^.

### Caspase-8 mediated NLRP3 inflammasome-induced IL-1β activation

Since caspase-8 activity leads to the processing of IL-1β and its secretion, TLR4-initiated RIPK3-mediated activation of caspase-8 activity in cIAP-depleted macrophages leads to the production of mature IL-1β ^146^. Perhaps more significantly, NLRP3 inflammasome-induced IL-1β activation by TLR4 requires RIPK3 along with ROS production^146,147^. In the absence of caspase-8, this requires MLKL, but in its presence, only the expression RIPK3 is required for inflammasome activation^148^. NLRP3 inflammasome activation occurs prior to or independently of necroptosis^146,147^. RIPK3 is also required for TLR3-mediated late signals that activate the inflammasome, which also has a corequirement for MLKL^149^. Therefore, RIPK3 can promote NLRP3 inflammasome and IL-1β inflammatory responses both dependent and independent of MLKL. In the absence of A20, LPS induces spontaneous NLRP3 inflammasome activation that is dependent on RIPK3^150^. In this case, the ubiquitylation of pro-IL-1β is increased, which then further promotes IL-1β cleavage and activation. Importantly, pathogens may thus engage RIPK3-mediated signaling to activate NLRP3^151^.

### NF-κB activation

Elucidating a role for RIPK3 in NF-κB activation has been a back-and-forth story. When first discovered, overexpression studies indicated that RIPK3 activated the NF-κB pathway^3,4,152^. Later, studies in cells from knockout mice concluded that it did not affect TNF-induced NF-κB^13,153^. However, these studies based their observations on IκBα phosphorylation and degradation, which were similar between WT and KO mice. Closer examination revealed that although RIPK3 did not affect IκBα, LPS-induced and NF-κB-dependent cytokine expression were greatly hampered in bone marrow dendritic cells^154^. Further examination revealed that nuclear translocation of the RelB-p50 heterodimer of NF-κB was impaired in RIPK3 knockout cells. Thus, noncanonical NF-κB activity requires RIPK3 in specific cell types.

### Metabolism

RIPK3 may have additional alternative roles in regulating metabolic enzymes associated with glycolysis and the mitochondria. Zhang et al. identified several metabolic enzymes in screening for interactions with RIPK3, including glycogen phosphorylase (PYGL), glutamate-ammonia ligase (GLUL), glutamate dehydrogenase 1 (GLUD1), as well as fructose-1,6-bisphosphatase 2 (FBP2), fumarate hydratase (FH), glycosyltransferase 25 domain-containing 1 (GLT25D1), and isocitrate dehydrogenase 1 (IDH1)^15^. The interaction of PYGL, GLUL, and GLUD1 with RIPK3 was verified in overexpression systems^15^. Later work by the same group further showed more convincingly that RIPK3 (and MLKL) activates the pyruvate dehydrogenase complex to increase aerobic respiration^102^. This acts as a source of ROS during necroptosis but may also regulate metabolism outside of a cell death setting.

## RIPK3 kinase activities

Very little has actually been studied with regard to RIPK3 substrates, other than those found in necroptosis. While there are some functions of RIPK3 (mostly adaptor complex functions) that can occur in the absence of kinase activity^155^, most RIPK3 activities are due to its enzyme function as a serine-threonine kinase. In necroptosis, it is largely the phosphorylation of the MLKL activation loop at T357, S358, S345, and S347 in human MLKL or T349 and S352 in mouse MLKL that is necessary for necroptosis to proceed. This activity is considered to be the standard canonical kinase function. However, MLKL is definitely not the only substrate for RIPK3. As mentioned in the preceding paragraph, RIPK3 phosphorylates PYGL, GLUL, GLUD1, and other metabolic enzymes mainly associated with mitochondrial metabolic pathways to increase aerobic respiration, which may or may not be solely associated with necroptosis^15,102^.

Interestingly, Al-Moujahed et al. showed that the deletion of RIPK3 suppresses the reprogramming of MEFs into induced pluripotent stem cells (iPSCs), a phenomenon that, in association with other data indicating that the growth rate of RIPK3 KO MEFs is significantly lower than that of WT MEFs, led them to conclude that this was because RIPK3 affects the expression of cell cycle/cell division genes. Consistent with this, phosphoproteomic analysis of possible RIPK3 phosphorylated peptides concluded that many of them were functionally associated with the cell cycle^156,157^. Therefore, while little is known about cell cycle-specific substrates, it is likely that RIPK3 has other functions of its kinase activity outside of necroptosis.

Among the more recently identified substrates of RIPK3 is the autophagy protein ULK1, which regulates both canonical and alternative autophagy^158^. In our 2015 paper, we found that cytotoxic chemotherapy, which induces DNA-damaging agents, induces RIPK1/RIPK3 activity and subsequent necroptosis^159^. Torri et al. found that RIPK1-independent RIPK3 phosphorylation is also induced by the genotoxic stress associated with DNA-damaging agents, and RIPK3 then phosphorylates ULK1 on S746. This phosphorylation event thereby activates alternative autophagy^158^.

## Diseases involving RIPK3

In our work in cancer cell lines, we found that necroptosis was induced by chemotherapeutics; further analysis revealed that RIPK3 was silenced by methylation in cancer cell lines and primary cancers, suggesting that the expression of necrotic cell death molecules may play a role in tumor repression and chemotherapy resistance in cancers. Other investigations have concluded that necroptosis/RIPK3 has a role in cancer mitigation and control^32,92,159–164^; it has also been suggested that necroptosis-mediated inflammation and cell death may alternatively contribute to tumorigenesis and an immunosuppressive tumor microenvironment^163,165–167^. As mentioned previously, it has later become evident that necroptosis may play significant roles in immunosurveillance due to the de novo synthesis of cytokines and chemokines that occurs especially in dying necroptotic cells^22–24^, along with the release of DAMPs^28^, which promote efficient immunogenic responses to cancer cells^25–28^.

**Fig. 2:**
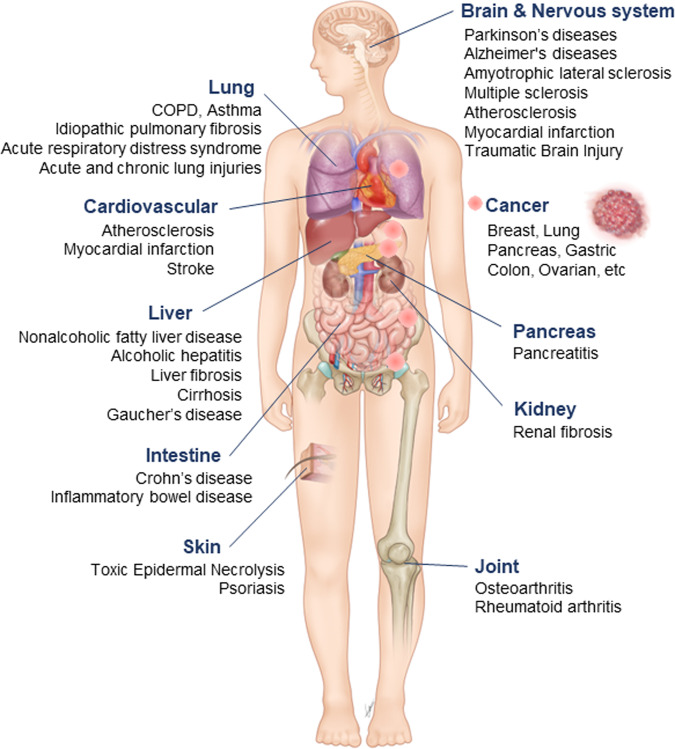
Impact of RIPK3-mediated necroptosis in human diseases. RIPK3-mediated necroptosis pathway dysregulation has been implicated in the pathophysiological processes of several human diseases, including various cancers and liver, cardiovascular, neurodegenerative, lung, pancreatic, intestinal, kidney, skin, and joint diseases.

Over time, the list of diseases that involve necroptosis and/or RIPK3 function has grown (see Fig. 2). Originally, this list included a facilitative role in tissue damage, such as in ischemia-reperfusion injury^11,168^, atherosclerosis^169,170^ and host defense against viral infections^171^.

The findings from recent research in cardiovascular diseases have continued to suggest roles for RIPK3/necroptosis^172^. Multiple cardiovascular pathologies are affected, including atherosclerosis, myocardial infarction (see, for instance, ref. ^173^), stroke and the accompanying (previously mentioned) ischemia-reperfusion injury, abdominal aortic aneurysm, myocarditis, and thrombosis^172,174,175^.

RIPK3/necroptosis is involved in lung disease and injury^176–178^. This includes acute respiratory distress syndrome and both acute and chronic lung injuries, both infectious and sterile in nature^177^. Pulmonary diseases in which necroptosis/RIPK3 plays a role are thought to include COPD, idiopathic pulmonary fibrosis, and asthma.

In the liver, RIPK3/necroptosis has a role in many pathological conditions^174,179–183^. Reports in which RIPK3/necroptosis is implicated include immune-mediated liver injury, nonalcoholic fatty liver disease (specifically nonalcoholic steatohepatitis), alcoholic hepatitis, liver fibrosis, and cirrhosis. RIPK3 may also play a role in Gaucher’s disease, which can have a heavy liver component^184^.

In kidney diseases, RIPK3/necroptosis has been reported to be involved in acute kidney injury and chronic kidney diseases, and the resulting renal fibrosis^176,185–187^.

Aside from the cardiovascular conditions previously mentioned above, RIPK3/necroptosis is implicated in many pathologies of the brain and nervous system. Among these are several neurodegenerative diseases, including Parkinson’s disease, Alzheimer’s disease, amyotrophic lateral sclerosis (ALS), and multiple sclerosis^174,176,188,189^. Targeting traumatic brain injury (TBI) through RIPK3 inhibition is also currently a matter of investigation^189^.

RIPK3 is thought to play a role in other autoimmune and inflammatory diseases of various organs, including the skin. We have shown a role for RIPK3 in toxic epidermal necrolysis (TEN)^190^, a condition of the skin and mucous membranes that results from adverse drug reactions. Among other inflammatory diseases, RIPK1/RIPK3 are believed to play a role in psoriasis (an autoimmune condition of the skin), rheumatoid arthritis (RA), pancreatitis, Crohn’s disease, and inflammatory bowel disease (IBD)^174,176,188^. In addition to rheumatoid arthritis, we have found that RIPK3 contributes to osteoarthritis (OA), through roles at least partly independent of MLKL activation^191^.

## Conclusion

There is clearly much work to be done in the necroptosis field with respect to cell death mechanisms and the involvement of RIPK3 in signaling and disease. Due to the putative effects on disease and inflammation, further efforts to understand the roles of RIPK3 in signaling and disease are likely to be of significant benefit to science in general and especially have implications for therapeutic gains in the treatment of human diseases.

## References

[1] Verma I (2002). 2002 Nobel Prize for physiology and medicine. Mol. Ther..

[2] Galluzzi L (2018). Molecular mechanisms of cell death: recommendations of the Nomenclature Committee on Cell Death 2018. Cell Death Differ..

[3] Sun X (1999). RIP3, a novel apoptosis-inducing kinase. J. Biol. Chem..

[4] Yu PW (1999). Identification of RIP3, a RIP-like kinase that activates apoptosis and NFkappaB. Curr. Biol..

[5] Kasof GM, Prosser JC, Liu D, Lorenzi MV, Gomes BC (2000). The RIP-like kinase, RIP3, induces apoptosis and NF-kappaB nuclear translocation and localizes to mitochondria. FEBS Lett..

[6] Grooten J, Goossens V, Vanhaesebroeck B, Fiers W (1993). Cell membrane permeabilization and cellular collapse, followed by loss of dehydrogenase activity: early events in tumour necrosis factor-induced cytotoxicity. Cytokine.

[7] Vercammen D (1998). Inhibition of caspases increases the sensitivity of L929 cells to necrosis mediated by tumor necrosis factor. J. Exp. Med..

[8] Holler N (2000). Fas triggers an alternative, caspase-8-independent cell death pathway using the kinase RIP as effector molecule. Nat. Immunol..

[9] Laster SM, Wood JG, Gooding LR (1988). Tumor necrosis factor can induce both apoptic and necrotic forms of cell lysis. J. Immunol..

[10] Denecker G (2001). Death receptor-induced apoptotic and necrotic cell death: differential role of caspases and mitochondria. Cell Death Differ..

[11] Degterev A (2005). Chemical inhibitor of nonapoptotic cell death with therapeutic potential for ischemic brain injury. Nat. Chem. Biol..

[12] Lin Y (2004). Tumor necrosis factor-induced nonapoptotic cell death requires receptor-interacting protein-mediated cellular reactive oxygen species accumulation. J. Biol. Chem..

[13] Cho YS (2009). Phosphorylation-driven assembly of the RIP1-RIP3 complex regulates programmed necrosis and virus-induced inflammation. Cell.

[14] He S (2009). Receptor interacting protein kinase-3 determines cellular necrotic response to TNF-alpha. Cell.

[15] Zhang DW (2009). RIP3, an energy metabolism regulator that switches TNF-induced cell death from apoptosis to necrosis. Science.

[16] Morgan M, Liu Z (2013). Programmed cell death with a necrotic-like phenotype. Biomol. Concepts.

[17] Vanlangenakker N, Vanden Berghe T, Vandenabeele P (2012). Many stimuli pull the necrotic trigger, an overview. Cell Death Differ..

[18] Kroemer G (2005). Classification of cell death: recommendations of the Nomenclature Committee on Cell Death. Cell Death Differ..

[19] Fiers W, Beyaert R, Declercq W, Vandenabeele P (1999). More than one way to die: apoptosis, necrosis and reactive oxygen damage. Oncogene.

[20] Kaczmarek A, Vandenabeele P, Krysko DV (2013). Necroptosis: the release of damage-associated molecular patterns and its physiological relevance. Immunity.

[21] Choi ME, Price DR, Ryter SW, Choi AMK (2019). Necroptosis: a crucial pathogenic mediator of human disease. JCI Insight.

[22] Gong Y-N (2017). ESCRT-III acts downstream of MLKL to regulate necroptotic cell death and its consequences. Cell.

[23] Zhu K (2018). Necroptosis promotes cell-autonomous activation of proinflammatory cytokine gene expression. Cell Death Dis..

[24] Orozco SL (2019). RIPK3 activation leads to cytokine synthesis that continues after loss of cell membrane integrity. Cell Rep..

[25] Yatim N (2015). RIPK1 and NF-kappaB signaling in dying cells determines cross-priming of CD8(+) T cells. Science.

[26] Snyder AG (2019). Intratumoral activation of the necroptotic pathway components RIPK1 and RIPK3 potentiates antitumor immunity. Sci. Immunol.

[27] Park HH (2021). RIPK3 activation induces TRIM28 derepression in cancer cells and enhances the anti-tumor microenvironment. Mol. Cancer.

[28] Aaes TL (2016). Vaccination with necroptotic cancer cells induces efficient anti-tumor immunity. Cell Rep..

[29] Zhao J (2012). Mixed lineage kinase domain-like is a key receptor interacting protein 3 downstream component of TNF-induced necrosis. Proc. Natl Acad. Sci. USA.

[30] Sun L (2012). Mixed lineage kinase domain-like protein mediates necrosis signaling downstream of RIP3 kinase. Cell.

[31] Wu JF (2013). Mlkl knockout mice demonstrate the indispensable role of Mlkl in necroptosis. Cell Res..

[32] Murphy JM (2013). The pseudokinase MLKL mediates necroptosis via a molecular switch mechanism. Immunity.

[33] Hildebrand JM (2014). Activation of the pseudokinase MLKL unleashes the four-helix bundle domain to induce membrane localization and necroptotic cell death. Proc. Natl Acad. Sci. USA.

[34] Li L, Tong A, Zhang Q, Wei Y, Wei X (2021). The molecular mechanisms of MLKL-dependent and MLKL-independent necrosis. J. Mol. Cell Biol..

[35] Chen W (2013). Diverse sequence determinants control human and mouse receptor interacting protein 3 (RIP3) and mixed lineage kinase domain-like (MLKL) interaction in necroptotic signaling. J. Biol. Chem..

[36] Petrie EJ (2018). Conformational switching of the pseudokinase domain promotes human MLKL tetramerization and cell death by necroptosis. Nat. Commun..

[37] Huang D (2017). The MLKL channel in necroptosis is an octamer formed by tetramers in a dyadic process. Mol. Cell Biol.

[38] Wang HY (2014). Mixed lineage kinase domain-like protein MLKL causes necrotic membrane disruption upon phosphorylation by RIP3. Mol. Cell.

[39] Dondelinger Y (2014). MLKL compromises plasma membrane integrity by binding to phosphatidylinositol phosphates. Cell Rep..

[40] Quarato G (2016). Sequential engagement of distinct MLKL phosphatidylinositol-binding sites executes necroptosis. Mol. Cell.

[41] Dovey CM (2018). MLKL requires the inositol phosphate code to execute necroptosis. Mol. Cell.

[42] Xia B (2016). MLKL forms cation channels. Cell Res..

[43] Ros U (2017). Necroptosis execution is mediated by plasma membrane nanopores independent of calcium. Cell Rep..

[44] Cai ZY (2014). Plasma membrane translocation of trimerized MLKL protein is required for TNF-induced necroptosis. Nat. Cell Biol..

[45] Chen X (2014). Translocation of mixed lineage kinase domain-like protein to plasma membrane leads to necrotic cell death. Cell Res.

[46] Vandenabeele P, Galluzzi L, Vanden Berghe T, Kroemer G (2010). Molecular mechanisms of necroptosis: an ordered cellular explosion. Nat. Rev. Mol. Cell Biol..

[47] Galluzzi L (2015). Essential versus accessory aspects of cell death: recommendations of the NCCD 2015. Cell Death Differ..

[48] Schock SN (2017). Induction of necroptotic cell death by viral activation of the RIG-I or STING pathway. Cell Death Differ..

[49] Robinson N (2012). Type I interferon induces necroptosis in macrophages during infection with Salmonella enterica serovar Typhimurium. Nat. Immunol..

[50] Brault M, Olsen TM, Martinez J, Stetson DB, Oberst A (2018). Intracellular nucleic acid sensing triggers necroptosis through synergistic type I IFN and TNF signaling. J. Immunol..

[51] Sun X, Yin J, Starovasnik MA, Fairbrother WJ, Dixit VM (2002). Identification of a novel homotypic interaction motif required for the phosphorylation of receptor-interacting protein (RIP) by RIP3. J. Biol. Chem..

[52] Devin A, Lin Y, Liu ZG (2003). The role of the death-domain kinase RIP in tumour-necrosis-factor-induced activation of mitogen-activated protein kinases. EMBO Rep..

[53] Degterev A (2008). Identification of RIP1 kinase as a specific cellular target of necrostatins. Nat. Chem. Biol..

[54] Petrie EJ, Czabotar PE, Murphy JM (2019). The structural basis of necroptotic cell death signaling. Trends Biochem. Sci..

[55] Li J (2012). The RIP1/RIP3 necrosome forms a functional amyloid signaling complex required for programmed necrosis. Cell.

[56] Mompean M (2018). The structure of the necrosome RIPK1-RIPK3 core, a human hetero-amyloid signaling complex. Cell.

[57] Orozco S (2014). RIPK1 both positively and negatively regulates RIPK3 oligomerization and necroptosis. Cell Death Differ..

[58] Lin Y, Devin A, Rodriguez Y, Liu ZG (1999). Cleavage of the death domain kinase RIP by caspase-8 prompts TNF-induced apoptosis. Genes Dev..

[59] Feng S (2007). Cleavage of RIP3 inactivates its caspase-independent apoptosis pathway by removal of kinase domain. Cell. Signal..

[60] O’Donnell MA (2011). Caspase 8 inhibits programmed necrosis by processing CYLD. Nat. Cell Biol..

[61] Morgan MJ, Liu ZG (2010). Reactive oxygen species in TNFalpha-induced signaling and cell death. Mol. Cells.

[62] Rickard JA (2014). RIPK1 regulates RIPK3-MLKL-driven systemic inflammation and emergency hematopoiesis. Cell.

[63] Dillon CP (2014). RIPK1 blocks early postnatal lethality mediated by caspase-8 and RIPK3. Cell.

[64] Dannappel M (2014). RIPK1 maintains epithelial homeostasis by inhibiting apoptosis and necroptosis. Nature.

[65] Bonnet MC (2011). The adaptor protein FADD protects epidermal keratinocytes from necroptosis in vivo and prevents skin inflammation. Immunity.

[66] Ch’en IL, Tsau JS, Molkentin JD, Komatsu M, Hedrick SM (2011). Mechanisms of necroptosis in T cells. J. Exp. Med.

[67] Dillon CP (2012). Survival function of the FADD-CASPASE-8-cFLIP(L) complex. Cell Rep..

[68] Kaiser WJ (2011). RIP3 mediates the embryonic lethality of caspase-8-deficient mice. Nature.

[69] Lu JV (2011). Complementary roles of Fas-associated death domain (FADD) and receptor interacting protein kinase-3 (RIPK3) in T-cell homeostasis and antiviral immunity. Proc. Natl Acad. Sci. USA.

[70] Moulin M (2012). IAPs limit activation of RIP kinases by TNF receptor 1 during development. EMBO J..

[71] Oberst A (2011). Catalytic activity of the caspase-8-FLIP(L) complex inhibits RIPK3-dependent necrosis. Nature.

[72] Zhang HB (2011). Functional complementation between FADD and RIP1 in embryos and lymphocytes. Nature.

[73] Pobezinskaya YL (2008). The function of TRADD in signaling through tumor necrosis factor receptor 1 and TRIF-dependent Toll-like receptors. Nat. Immunol..

[74] Ermolaeva MA (2008). Function of TRADD in tumor necrosis factor receptor 1 signaling and in TRIF-dependent inflammatory responses. Nat. Immunol..

[75] Chen NJ (2008). Beyond tumor necrosis factor receptor: TRADD signaling in toll-like receptors. Proc. Natl Acad. Sci. USA.

[76] Haas TL (2009). Recruitment of the linear ubiquitin chain assembly complex stabilizes the TNF-R1 signaling complex and is required for TNF-mediated gene induction. Mol. Cell.

[77] Fullsack S, Rosenthal A, Wajant H, Siegmund D (2019). Redundant and receptor-specific activities of TRADD, RIPK1 and FADD in death receptor signaling. Cell Death Dis..

[78] Seo J, Nam YW, Kim S, Oh DB, Song J (2021). Necroptosis molecular mechanisms: recent findings regarding novel necroptosis regulators. Exp. Mol. Med..

[79] Wertz IE (2004). De-ubiquitination and ubiquitin ligase domains of A20 downregulate NF-kappaB signalling. Nature.

[80] Wright A (2007). Regulation of early wave of germ cell apoptosis and spermatogenesis by deubiquitinating enzyme CYLD. Dev. Cell.

[81] Heger K (2018). OTULIN limits cell death and inflammation by deubiquitinating LUBAC. Nature.

[82] Annibaldi A (2018). Ubiquitin-mediated regulation of RIPK1 kinase activity independent of IKK and MK2. Mol. Cell.

[83] Chen M (2020). Smac mimetic promotes TNF-alpha to induce apoptosis of gallbladder carcinoma cells. Cell. Signal..

[84] Dondelinger Y (2019). Serine 25 phosphorylation inhibits RIPK1 kinase-dependent cell death in models of infection and inflammation. Nat. Commun..

[85] Xu D (2018). TBK1 suppresses RIPK1-driven apoptosis and inflammation during development and in aging. Cell.

[86] Tenev T (2011). The Ripoptosome, a signaling platform that assembles in response to genotoxic stress and loss of IAPs. Mol. Cell.

[87] Feoktistova M (2011). cIAPs block Ripoptosome formation, a RIP1/caspase-8 containing intracellular cell death complex differentially regulated by cFLIP isoforms. Mol. Cell.

[88] Amin P (2018). Regulation of a distinct activated RIPK1 intermediate bridging complex I and complex II in TNFalpha-mediated apoptosis. Proc. Natl Acad. Sci. USA.

[89] Wang H (2017). PELI1 functions as a dual modulator of necroptosis and apoptosis by regulating ubiquitination of RIPK1 and mRNA levels of c-FLIP. Proc. Natl Acad. Sci. USA.

[90] Seo J (2016). CHIP controls necroptosis through ubiquitylation- and lysosome-dependent degradation of RIPK3. Nat. Cell Biol..

[91] Choi SW (2018). PELI1 selectively targets kinase-active RIP3 for ubiquitylation-dependent proteasomal degradation. Mol. Cell.

[92] Morgan MJ, Kim YS (2015). The serine threonine kinase RIP3: lost and found. BMB Rep..

[93] Seong D (2020). Identification of MYC as an antinecroptotic protein that stifles RIPK1-RIPK3 complex formation. Proc. Natl Acad. Sci. USA.

[94] Xie Y (2017). Inhibition of aurora kinase A induces necroptosis in pancreatic carcinoma. Gastroenterology.

[95] Chen W (2015). Ppm1b negatively regulates necroptosis through dephosphorylating Rip3. Nat. Cell Biol..

[96] Lee SY (2019). Casein kinase-1gamma1 and 3 stimulate tumor necrosis factor-induced necroptosis through RIPK3. Cell Death Dis..

[97] Hanna-Addams S, Liu S, Liu H, Chen S, Wang Z (2020). CK1alpha, CK1delta, and CK1epsilon are necrosome components which phosphorylate serine 227 of human RIPK3 to activate necroptosis. Proc. Natl Acad. Sci. USA.

[98] Kim YS, Morgan MJ, Choksi S, Liu ZG (2007). TNF-induced activation of the Nox1 NADPH oxidase and its role in the induction of necrotic cell death. Mol. Cell.

[99] Yazdanpanah B (2009). Riboflavin kinase couples TNF receptor 1 to NADPH oxidase. Nature.

[100] Vanden Berghe T (2010). Necroptosis, necrosis and secondary necrosis converge on similar cellular disintegration features. Cell Death Differ..

[101] Goossens V, Grooten J, De Vos K, Fiers W (1995). Direct evidence for tumor necrosis factor-induced mitochondrial reactive oxygen intermediates and their involvement in cytotoxicity. Proc. Natl Acad. Sci. USA.

[102] Yang Z (2018). RIP3 targets pyruvate dehydrogenase complex to increase aerobic respiration in TNF-induced necroptosis. Nat. Cell Biol..

[103] Tait SW (2013). Widespread mitochondrial depletion via mitophagy does not compromise necroptosis. Cell Rep..

[104] Zhang Y (2017). RIP1 autophosphorylation is promoted by mitochondrial ROS and is essential for RIP3 recruitment into necrosome. Nat. Commun..

[105] Paulsen CE, Carroll KS (2010). Orchestrating redox signaling networks through regulatory cysteine switches. ACS Chem. Biol..

[106] Groen A (2005). Differential oxidation of protein-tyrosine phosphatases. J. Biol. Chem..

[107] Nakashima I (2002). Redox-linked signal transduction pathways for protein tyrosine kinase activation. Antioxid. Redox Signal..

[108] Nakashima I (2005). Redox control of catalytic activities of membrane-associated protein tyrosine kinases. Arch. Biochem. Biophys..

[109] Kamata H (2005). Reactive oxygen species promote TNFalpha-induced death and sustained JNK activation by inhibiting MAP kinase phosphatases. Cell.

[110] Yeh WC (1998). FADD: essential for embryo development and signaling from some, but not all, inducers of apoptosis. Science.

[111] Lawrence CP, Chow SC (2005). FADD deficiency sensitises Jurkat T cells to TNF-alpha-dependent necrosis during activation-induced cell death. FEBS Lett..

[112] Geserick P (2009). Cellular IAPs inhibit a cryptic CD95-induced cell death by limiting RIP1 kinase recruitment. J. Cell Biol..

[113] Vanlangenakker N (2011). cIAP1 and TAK1 protect cells from TNF-induced necrosis by preventing RIP1/RIP3-dependent reactive oxygen species production. Cell Death Differ..

[114] Bohgaki T (2011). Caspase-8 inactivation in T cells increases necroptosis and suppresses autoimmunity in Bim-/- mice. J. Cell Biol..

[115] Dondelinger Y (2013). RIPK3 contributes to TNFR1-mediated RIPK1 kinase-dependent apoptosis in conditions of cIAP1/2 depletion or TAK1 kinase inhibition. Cell Death Differ..

[116] Morioka S (2009). TAK1 kinase determines TRAIL sensitivity by modulating reactive oxygen species and cIAP. Oncogene.

[117] Goodall ML (2016). The autophagy machinery controls cell death switching between apoptosis and necroptosis. Dev. Cell.

[118] Sprick MR (2000). FADD/MORT1 and caspase-8 are recruited to TRAIL receptors 1 and 2 and are essential for apoptosis mediated by TRAIL receptor 2. Immunity.

[119] Kuang AA, Diehl GE, Zhang J, Winoto A (2000). FADD is required for DR4- and DR5-mediated apoptosis: lack of trail-induced apoptosis in FADD-deficient mouse embryonic fibroblasts. J. Biol. Chem..

[120] Peter ME (2000). The TRAIL DISCussion: It is FADD and caspase-8!. Cell Death Differ..

[121] Jouan-Lanhouet S (2012). TRAIL induces necroptosis involving RIPK1/RIPK3-dependent PARP-1 activation. Cell Death Differ..

[122] Meurette O (2007). TRAIL induces receptor-interacting protein 1-dependent and caspase-dependent necrosis-like cell death under acidic extracellular conditions. Cancer Res..

[123] Henry CM, Martin SJ (2017). Caspase-8 acts in a non-enzymatic role as a scaffold for assembly of a pro-inflammatory “FADDosome” complex upon TRAIL stimulation. Mol. Cell.

[124] Cao X, Pobezinskaya YL, Morgan MJ, Liu ZG (2011). The role of TRADD in TRAIL-induced apoptosis and signaling. FASEB J..

[125] Kim JY (2011). TRADD is critical for resistance to TRAIL-induced cell death through NF-kappaB activation. FEBS Lett..

[126] Lafont E (2017). The linear ubiquitin chain assembly complex regulates TRAIL-induced gene activation and cell death. EMBO J..

[127] Simoes Eugenio M (2021). TRIM21, a new component of the TRAIL-induced endogenous necrosome complex. Front. Mol. Biosci..

[128] Jin Z, El-Deiry WS (2006). Distinct signaling pathways in TRAIL- versus tumor necrosis factor-induced apoptosis. Mol. Cell Biol..

[129] Varfolomeev E (2005). Molecular determinants of kinase pathway activation by Apo2 ligand/tumor necrosis factor-related apoptosis-inducing ligand. J. Biol. Chem..

[130] He S, Liang Y, Shao F, Wang X (2011). Toll-like receptors activate programmed necrosis in macrophages through a receptor-interacting kinase-3-mediated pathway. Proc. Natl Acad. Sci. USA.

[131] Lim J (2019). Autophagy regulates inflammatory programmed cell death via turnover of RHIM-domain proteins. Elife.

[132] Rebsamen M, Meylan E, Curran J, Tschopp J (2008). The antiviral adaptor proteins Cardif and Trif are processed and inactivated by caspases. Cell Death Differ..

[133] McComb S (2012). cIAP1 and cIAP2 limit macrophage necroptosis by inhibiting Rip1 and Rip3 activation. Cell Death Differ..

[134] McComb S (2014). Cathepsins limit macrophage necroptosis through cleavage of Rip1 kinase. J. Immunol..

[135] Ariana A (2020). Tristetraprolin regulates necroptosis during tonic Toll-like receptor 4 (TLR4) signaling in murine macrophages. J. Biol. Chem..

[136] Kaiser WJ (2013). Toll-like receptor 3-mediated necrosis via TRIF, RIP3, and MLKL. J. Biol. Chem..

[137] Bernard JJ (2012). Ultraviolet radiation damages self noncoding RNA and is detected by TLR3. Nat. Med..

[138] Weber A (2010). Proapoptotic signalling through Toll-like receptor-3 involves TRIF-dependent activation of caspase-8 and is under the control of inhibitor of apoptosis proteins in melanoma cells. Cell Death Differ..

[139] Vercammen E, Staal J, Beyaert R (2008). Sensing of viral infection and activation of innate immunity by toll-like receptor 3. Clin. Microbiol. Rev..

[140] Jiao H (2020). Z-nucleic-acid sensing triggers ZBP1-dependent necroptosis and inflammation. Nature.

[141] Upton JW, Kaiser WJ, Mocarski ES (2012). DAI/ZBP1/DLM-1 complexes with RIP3 to mediate virus-induced programmed necrosis that is targeted by murine cytomegalovirus vIRA. Cell Host Microbe.

[142] Maelfait J (2017). Sensing of viral and endogenous RNA by ZBP1/DAI induces necroptosis. EMBO J..

[143] Nogusa S (2016). RIPK3 activates parallel pathways of MLKL-driven necroptosis and FADD-mediated apoptosis to protect against influenza A virus. Cell Host Microbe.

[144] Lin J (2016). RIPK1 counteracts ZBP1-mediated necroptosis to inhibit inflammation. Nature.

[145] Newton K (2016). RIPK1 inhibits ZBP1-driven necroptosis during development. Nature.

[146] Vince JE (2012). Inhibitor of apoptosis proteins limit RIP3 kinase-dependent interleukin-1 activation. Immunity.

[147] Kang TB, Yang SH, Toth B, Kovalenko A, Wallach D (2013). Caspase-8 blocks kinase RIPK3-mediated activation of the NLRP3 inflammasome. Immunity.

[148] Lawlor KE (2015). RIPK3 promotes cell death and NLRP3 inflammasome activation in the absence of MLKL. Nat. Commun..

[149] Kang S (2015). Caspase-8 scaffolding function and MLKL regulate NLRP3 inflammasome activation downstream of TLR3. Nat. Commun..

[150] Duong BH (2015). A20 restricts ubiquitination of pro-interleukin-1beta protein complexes and suppresses NLRP3 inflammasome activity. Immunity.

[151] Speir M, Lawlor KE (2021). RIP-roaring inflammation: RIPK1 and RIPK3 driven NLRP3 inflammasome activation and autoinflammatory disease. Semin. Cell Dev. Biol..

[152] Pazdernik NJ, Donner DB, Goebl MG, Harrington MA (1999). Mouse receptor interacting protein 3 does not contain a caspase-recruiting or a death domain but induces apoptosis and activates NF-kappaB. Mol. Cell Biol..

[153] Newton K, Sun XQ, Dixit VM (2004). Kinase RIP3 is dispensable for normal NF-KBs, signaling by the B-cell and T-cell receptors, tumor necrosis factor receptor 1, and toll-like receptors 2 and 4. Mol. Cell Biol..

[154] Moriwaki K (2014). The necroptosis adaptor RIPK3 promotes injury-induced cytokine expression and tissue repair. Immunity.

[155] Orozco S, Oberst A (2017). RIPK3 in cell death and inflammation: the good, the bad, and the ugly. Immunol. Rev..

[156] Wu X (2012). Investigation of receptor interacting protein (RIP3)-dependent protein phosphorylation by quantitative phosphoproteomics. Mol. Cell Proteom..

[157] Al-Moujahed A (2019). Receptor interacting protein kinase 3 (RIP3) regulates iPSCs generation through modulating cell cycle progression genes. Stem Cell Res.

[158] Torii S (2020). Identification of a phosphorylation site on Ulk1 required for genotoxic stress-induced alternative autophagy. Nat. Commun..

[159] Koo G-B (2015). Methylation-dependent loss of RIP3 expression in cancer represses programmed necrosis in response to chemotherapeutics. Cell Res..

[160] Colbert LE (2013). Pronecrotic mixed lineage kinase domain-like protein expression is a prognostic biomarker in patients with early-stage resected pancreatic adenocarcinoma. Cancer.

[161] Ertao Z (2016). Prognostic value of mixed lineage kinase domain-like protein expression in the survival of patients with gastric caner. Tumour Biol..

[162] Park S (2009). The receptor interacting protein 1 inhibits p53 induction through NF-kappaB activation and confers a worse prognosis in glioblastoma. Cancer Res.

[163] Seifert L (2016). The necrosome promotes pancreatic oncogenesis via CXCL1 and Mincle-induced immune suppression. Nature.

[164] Conev NV (2019). RIPK3 expression as a potential predictive and prognostic marker in metastatic colon cancer. Clin. Invest. Med..

[165] Wang W (2018). RIP1 kinase drives macrophage-mediated adaptive immune tolerance in pancreatic cancer. Cancer Cell.

[166] Jiao D (2018). Necroptosis of tumor cells leads to tumor necrosis and promotes tumor metastasis. Cell Res..

[167] Strilic B (2016). Tumour-cell-induced endothelial cell necroptosis via death receptor 6 promotes metastasis. Nature.

[168] Linkermann A (2013). Two independent pathways of regulated necrosis mediate ischemia-reperfusion injury. Proc. Natl Acad. Sci. USA.

[169] Lin J (2013). A role of RIP3-mediated macrophage necrosis in atherosclerosis development. Cell Rep..

[170] Meng L, Jin W, Wang X (2015). RIP3-mediated necrotic cell death accelerates systematic inflammation and mortality. Proc. Natl Acad. Sci. USA.

[171] Kaiser WJ, Upton JW, Mocarski ES (2013). Viral modulation of programmed necrosis. Curr. Opin. Virol..

[172] DeRoo E, Zhou T, Liu B (2020). The role of RIPK1 and RIPK3 in cardiovascular disease. Int. J. Mol. Sci..

[173] Piamsiri C, Maneechote C, Siri-Angkul N, Chattipakorn SC, Chattipakorn N (2021). Targeting necroptosis as therapeutic potential in chronic myocardial infarction. J. Biomed. Sci..

[174] Khoury MK, Gupta K, Franco SR, Liu B (2020). Necroptosis in the pathophysiology of disease. Am. J. Pathol..

[175] Leng Y (2021). Receptor interacting protein kinases 1/3: the potential therapeutic target for cardiovascular inflammatory diseases. Front. Pharmacol..

[176] Dai W, Cheng J, Leng X, Hu X, Ao Y (2021). The potential role of necroptosis in clinical diseases (Review). Int. J. Mol. Med.

[177] Faust H, Mangalmurti NS (2020). Collateral damage: necroptosis in the development of lung injury. Am. J. Physiol. Lung Cell Mol. Physiol..

[178] Wang L (2021). Necroptosis in pulmonary diseases: a new therapeutic target. Front. Pharmacol..

[179] Dara L (2018). The receptor interacting protein kinases in the liver. Semin. Liver Dis..

[180] Gautheron J, Gores GJ, Rodrigues CMP (2020). Lytic cell death in metabolic liver disease. J. Hepatol..

[181] Li X, Dong G, Xiong H, Diao H (2021). A narrative review of the role of necroptosis in liver disease: a double-edged sword. Ann. Transl. Med..

[182] Saeed WK, Jun DW, Jang K, Koh DH (2019). Necroptosis signaling in liver diseases: an update. Pharmacol. Res..

[183] Shojaie L, Iorga A, Dara L (2020). Cell death in liver diseases: a review. Int. J. Mol. Sci.

[184] Yanez MJ (2021). c-Abl activates RIPK3 signaling in Gaucher disease. Biochim. Biophys. Acta Mol. Basis Dis..

[185] Anders HJ (2018). Necroptosis in acute kidney injury. Nephron.

[186] Shi Y, Chen X, Huang C, Pollock C (2020). RIPK3: a new player in renal fibrosis. Front. Cell Dev. Biol..

[187] Uni R, Choi ME (2021). Novel roles of necroptosis mediator receptor-interacting protein kinase 3 in kidney injury. Nephron.

[188] Khan I, Yousif A, Chesnokov M, Hong L, Chefetz I (2021). A decade of cell death studies: breathing new life into necroptosis. Pharmacol. Ther..

[189] Yu Z, Jiang N, Su W, Zhuo Y (2021). Necroptosis: a novel pathway in neuroinflammation. Front. Pharmacol..

[190] Kim SK (2015). Upregulated RIP3 expression potentiates MLKL phosphorylation-mediated programmed necrosis in toxic epidermal necrolysis. J. Invest. Dermatol..

[191] Jeon J (2020). TRIM24-RIP3 axis perturbation accelerates osteoarthritis pathogenesis. Ann. Rheum. Dis..

